# 3D gel dosimeter assessment for end-to-end geometric accuracy determination of the online adaptive workflow on the 1.5 T MR-linac

**DOI:** 10.1016/j.phro.2024.100664

**Published:** 2024-11-05

**Authors:** Stijn Oolbekkink, Jochem W.H. Wolthaus, Bram van Asselen, Bas W. Raaymakers

**Affiliations:** Department of Radiotherapy, University Medical Center Utrecht, Heidelberglaan 100, 3584 CX, Utrecht, the Netherlands

**Keywords:** Adaptive radiotherapy, MRI-linac, Quality assurance, End-to-end testing, 3D gel dosimetry

## Abstract

**Background and purpose:**

During an end-to-end (E2E) test on the online workflow of the MR-linac, the performance of the treatment starting from the acquisition of pre-treatment MRI scans and ending with dose delivery is quantified. In such a test, the geometrical accuracy of the entire workflow is assessed. Ideally, the 3D geometrical accuracy of dose delivery on an MR-linac should be assessed using dosimeters that provide 3D dose distributions. Gel dosimeters, for instance, have proven to be valuable tools for evaluating 3D dose distributions on an MR-linac. In this study, we investigated the use of 3D gel dosimeters for the assessment of the 3D geometrical accuracy and reproducibility of the adaptive procedure on an MR-linac in an E2E verification.

**Materials and methods:**

All measurements were performed on a clinical Unity MR-linac using 3D gel dosimeters in an anthropomorphic head phantom. Film measurements were performed as a reference dosimeter. An online adapt-to-shape procedure was performed for each measurement.

**Results:**

The geometric accuracy and reproducibility of the gel dosimeter measurements were high, and similar to all in-plane film measurements. The largest shift found was 0.3 mm for the gel dosimeter, and 0.6 mm for the in-plane film measurements. The 3D displacement vectors of the gel dosimeter showed similar uncertainties as the in-plane film 2D displacement vectors.

**Conclusions:**

Gel dosimeters can be used for the assessment of the 3D end-to-end geometric accuracy of an MR-linac.

## Highlights

• Gel dosimeters can be used for 3D geometric end-to-end accuracy testing on the MR-linac.
• Gel dosimeters showed similar performance to in-plane film dosimetry measurements.
• The end-to-end geometrical accuracy of the online adapt-to-shape procedure was high.
• The reproducibility of the online adapt-to-shape procedure was high.

## 1 Introduction

The addition of a MRI scanner to a linear accelerator, an MR-linac, gives high-quality soft tissue contrast images which can be used to correct the impact of anatomical changes before and during treatment [1], [2], [3], [4]. Various online adaptive workflows for the MR-linac have been developed [5]. The geometric and dosimetric accuracy of these adaptive online workflows can be assessed with an end-to-end (E2E) test involving all steps of the clinical workflow [6].

In an E2E test for a conventional linac, the performance of the treatment workflow starting from the acquisition of planning CTs or MRIs and ending with dose delivery is quantified [7], [8], [9]. The main steps involve image acquisition, image registration, delineation, treatment planning, position verification and dose delivery. For an MR-linac, the pre-treatment information is still required to start the online workflow, subsequently proceeding with the same steps as considered conventionally [7].

The overall treatment accuracy reported in literature mostly focuses on the dosimetric accuracy [10], [11], [12]. The dosimetric accuracy is commonly assessed via several metrics such as dose-volume histograms, gamma analyses, and dose profile comparisons. The general consensus is that the dosimetric accuracy of the online adaptive procedures on MR-linacs is high [10], [11], [12]. However, as the intended dose should be delivered at the right spot, the geometrical accuracy of the delivered dose, is just as important.

During the first-in-man study of the Unity MR-linac [4], the geometrical accuracy of the complete workflow was investigated on a phantom as well as in-vivo in patients using the electronic portal imaging device (EPID). Raaymakers et al. found that the geometric accuracy was better than 0.5 mm. During this study, the assessment of the 3D geometric accuracy required analysis of multiple beam projections. In a more recent study, Bernchou et al. investigated the geometrical accuracy of the adaptive procedures on MR-linacs using film dosimetry [13]. During their study, multiple workflows were performed using two orthogonal measurement planes to determine the geometric accuracy in 3D.

Ideally, the 3D geometrical accuracy of the dose delivery on an MR-linac should be determined using dosimeters which provide 3D dose distributions, for example using gel dosimeters [14], [15]. Previous studies have investigated the use of gel dosimeters for an MR-linac, showing their suitability in an MR-linac [16], [17], [18], [19], [20]. This is particularly beneficial for plan delivery assessment of complex, multi, out-of-plane targets, providing a high spatial comparison between the planned and measured doses in 3D, rather than a 2D plane or a single-point comparison [16]. The 3D dose distribution can potentially help determine the geometrical accuracy in an E2E test without needing multiple beam projections or orthogonal planes.

In this study, we investigate the use of gel dosimeters for the assessment of the 3D geometrical E2E accuracy of the adaptive procedure on an MR-linac.

**Fig. 1:**
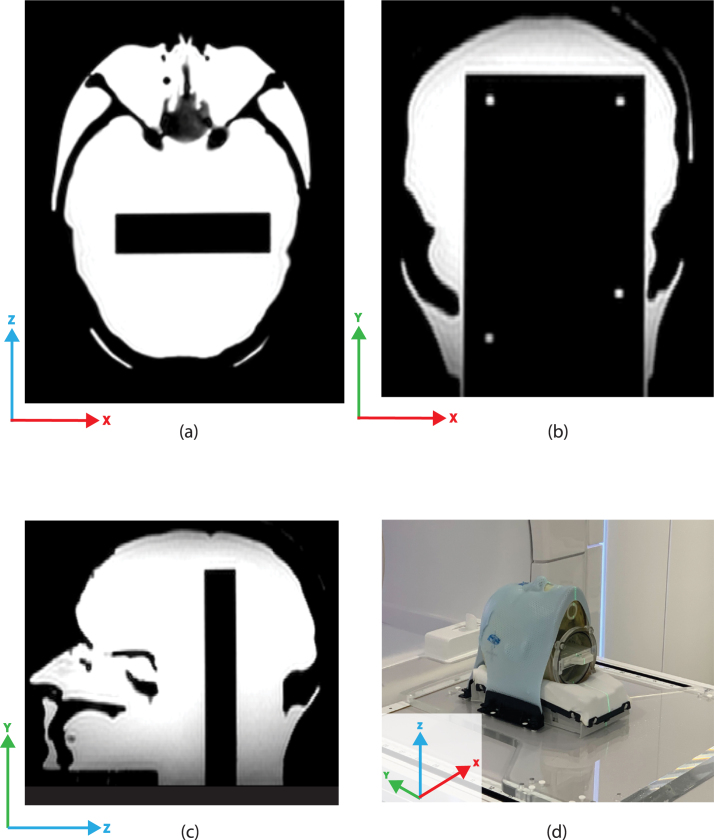
MR images of the Prime head phantom with the film insert in transversal, coronal and sagittal plane. The cradle and mask were mounted on the base plate. The body coil is positioned centrally over the Prime head phantom.

## 2 Materials and methods

During this study, the usability of gel dosimeters for determination of the geometrical accuracy in an E2E test was assessed for the online adapt-to-shape (ATS) workflow of the Unity MR-linac (Elekta AB, Stockholm, Sweden) [5]. The complete clinical workflow, involving pre-treatment plan and online adapted plan generation was measured with a gel dosimeter in an anthropomorphic head phantom. Film measurements were performed using two measurement plane orientations as a reference.

### 2.1 Phantom setup

All measurements were performed on the same Unity MR-linac to avoid calibration and machine differences. Measurements were carried out using the Prime head phantom (RTsafe, Athens, Greece), which has anthropomorphic features such as nasal cavities based on CT images of a human head (Fig. 1a–c) [16], [21], [22]. Throughout this paper, the IEC 61217 treatment coordinate system is used [23].

The Prime phantom was positioned using an index bar on which an Exafix 5 base plate (Macromedics, Moordrecht, the Netherlands) carrying a custom-shaped headrest (Civco, Iowa, USA) and head mask (Macromedics, Moordrecht, the Netherlands) was fixated. This setup minimized the effect of positioning variations [24]. Two inserts for the Prime phantom were used: the gel and film inserts. After measuring with the gel insert, it was replaced by the film insert, the head was refilled with water, and remounted using the mask system.

### 2.2 Pre-treatment workflow

Pre-treatment CT and MRI scans of the Prime phantom were acquired for the setup with a gel insert and one with the film insert oriented horizontally (coronal measurement plane) using a big bore RT CT scanner (Philips Healthcare, Best, the Netherlands), and an Ingenia 1.5 T MRI (Philips Healthcare, Best, the Netherlands) using the head coil. Images were acquired using a T2-weighted sequence with a pixel size of 0.7 × 0.7 ${\mathrm{mm}}^{2}$ and a slice thickness of 1.1 mm (FA=90°, TE=332 ms, TR=2100 ms, BW=813 Hz/px).

**Fig. 2:**
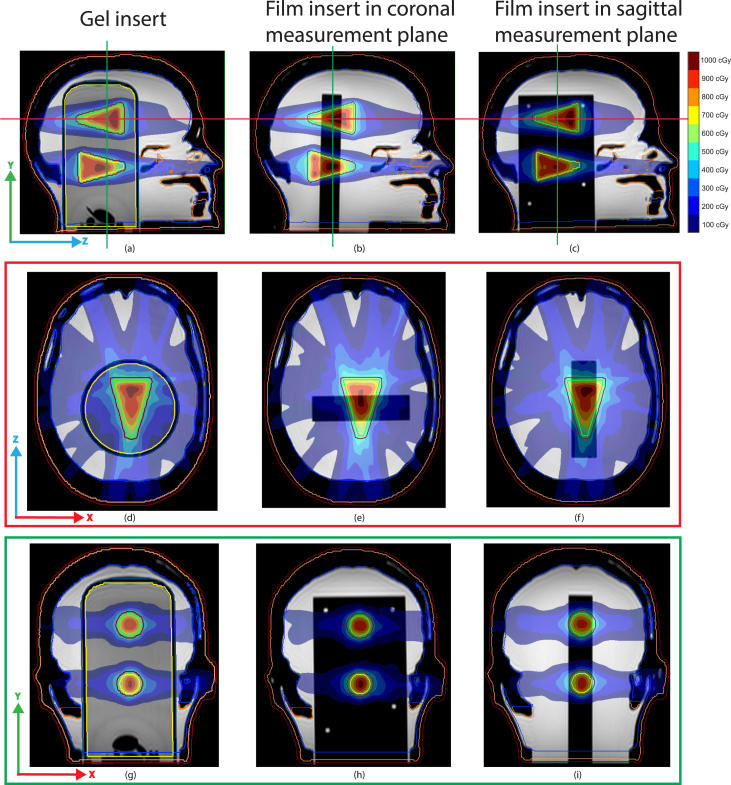
The pre-treatment planned dose with the unambiguous PTVs used for both the gel and the film inserts. The sagittal pre-treatment planned dose for the Prime phantom with the gel (a) and film inserts (b (coronal) and c (sagittal)). The transversal pre-treatment dose for PTV 1 is shown in d (gel), e (film) and f (film). The coronal planned pre-treatment dose shown in g, h and i for the gel and film inserts The green and red lines in a, b and c schematically indicate the locations of the d, e, f and, g, h, i images. For the sagittal orientation of the film insert the dose distribution is simulated. (For interpretation of the references to colour in this figure legend, the reader is referred to the web version of this article.)

Several contours like the body, internal bony structures, water, and the base plate were created using the treatment planning system (TPS) Monaco (v.5.51.10, Elekta AB, Stockholm, Sweden) as well as two cones that serve as planning target volumes (PTVs). The two PTVs were identical but mirrored with respect to each other in the z-direction (see Fig. 2). The smallest and largest diameters were 0.5 cm and 3.0 cm, respectively, with a length of 4.6 cm. Due to the design of the PTVs, geometric uncertainties orthogonal to the 2D film measurement plane could be assessed. The PTVs were positioned to ensure that the high-dose regions, with a maximum dose of 11 Gy, were measurable in both gel and film dosimeters. Additionally, a maximum dose constraint of 6 Gy was applied to the head, excluding the PTVs. Electron densities (ED) were manually assigned to the delineated structures based on CT-values. ED-values for the skull and its internal bony structures were set to 1.550. The gel and glass cylinder insert ED-values were set to 1.147 and 1.650, respectively. The film insert had an assigned ED value of 1. The same treatment plan optimization constraints were applied to the pre-treatment plans of both gel and film inserts, ensuring similar dose distribution in the PTVs for both dosimeters. The plan’s isocenter was defined using the Prime phantom’s ears and was located near PTV 2. An IMRT plan was created to irradiate both PTVs using a step-and-shoot technique with thirteen beams; 190°, 220°, 250°, 280°, 310°, 340°, 5°, 20°, 50°, 80°, 110°, 140°, and 170°. For plan generation, a maximum of 150 segments with a minimum segment area of 1.5 ${\mathrm{cm}}^{2}$ and a minimum of 5 MU per segment was used.

### 2.3 Online workflow

Online workflows were performed using the ATS plan optimization from fluence using the Monaco TPS (v.5.51.10 and v.6.1.2) [5]. Online MR images were acquired using the built-in MR system of the MR-linac using the standard patient setup, employing the same sequence used for the pre-treatment MRI acquisitions. The online images were rigidly registered by the Monaco TPS to the pre-treatment MRI. As the Prime phantom might be positioned slightly different in the pre-treatment scan, small differences were expected. After registration, the PTV structures and baseplate were rigidly propagated from the pre-treatment plan to the online plan. The body, internal bony structures, and water contours were propagated deformably, simulating the clinical workflow. Subsequently, the online treatment plan was generated, and the dose was calculated using a voxel size of 3.0 × 3.0 × 3.0 ${\mathrm{mm}}^{3}$ and 3% statistical uncertainty per segment.

For comparison to the measured data, the treatment plan after treatment was recalculated using a voxel size of 1.0 × 1.0 × 1.0 ${\mathrm{mm}}^{3}$ and a statistical uncertainty of 0.5% per control point to avoid discretization errors and statistical uncertainty on the dose calculation. This resulted in an overall statistical uncertainty of the total plan dose of approximately 0.1% inside of the PTVs. The recalculation was performed after the measurements in an offline setting.

### 2.4 Experiments

To assess the performance of gel dosimeters during 3D geometric E2E accuracy determination, two measurement sessions were performed. For each measurement, an online ATS workflow was performed. In the first session, 3D gel measurements (see Section 2.5) were compared to 2D film (coronal measurement plane) measurements (see Section 2.6). In the second session, film measurements were performed in both coronal and sagittal insert orientations. Each session involved five measurements, over five different days within a two week period.

### 2.5 Gel dosimetry

**Fig. 3:**
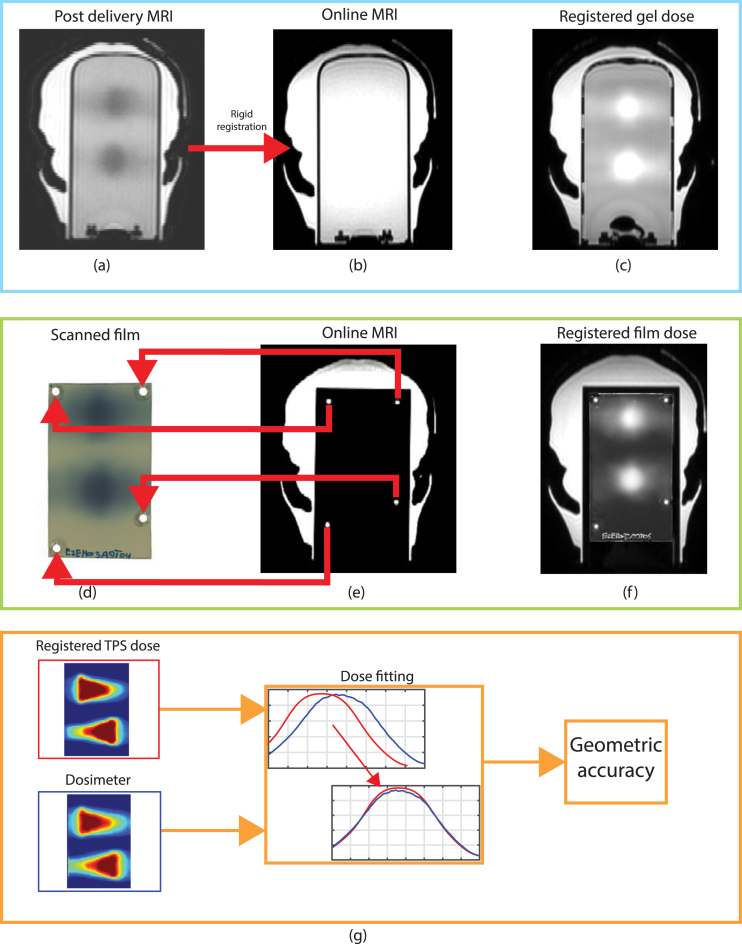
Registration workflow of the gel (a–c) and film dosimeter (d–f) to the online MRI. The post delivery MRI (obtained on a different scanner) is rigidly registered to the MRI. The registration of the gel dosimeter dose and the online MRI is shown in (c). The film registration is performed using the markers found in the online MRI (d) to the film (e). The registered film dose and online MRI is shown in (f). The workflow used to obtain the geometric accuracy between the registered TPS dose and the dosimeter is shown schematically in (g).

3D dosimetry was performed using N-vinylpyrrolidone argon (VIPAR) gels, manufactured by RTsafe (Athens, Greece), which were encapsulated in glass cylinders of 160 mm in height and 80 mm in diameter [14]. These gels dose exhibit linearity up to 20 Gy and were suitable for dosimetry in an MR-linac [16], [25], [26]. The gels were handled according to the manufacturer’s guidelines: A MRI scan of the Prime phantom with the gel insert was performed approximately 24 h after dose delivery on a 1.5 T MRI scanner using a head coil, employing a scan protocol (with a pixel size of 0.78 × 0.78 ${\mathrm{mm}}^{2}$ and a slice thickness of 2 mm) provided by RTsafe (Fig. 3a). The acquired MR images were processed by the RTsafe. In this process, the post-delivery MRI scan was registered to the online MRI scan of the ATS workflow (Figs. 3a and 3b) followed by reconstruction of the measured R2 relaxation rate maps [14], [27], [28]. These reconstructed R2 maps were then linearly converted to dose, and scaled with the maximum calculated dose. After processing, a 3D (normalized) dose grid with a voxel size of 1.0 × 1.0 × 1.0 ${\mathrm{mm}}^{3}$ was obtained.

### 2.6 Film dosimetry

EBT3 GafChromic (Ashland, New Jersey, USA) film was used for 2D dosimetry with the film insert in the Prime phantom [29]. The film cassette used was a modified version of the film cassette described by E. Pappas et al. [16]. In our version, the markers were replaced with hollow cylinders, capped at one end, allowing ultrasound gel to be inserted prior to the measurements and thereby increasing the MRI contrast from the marker on MR images. The film was securely held in place within the cassette via circular cut-outs in the film, to ensure a fixed position. During the film measurements water used to fill the small gaps between the film cassette and the film to minimize the effect of air pockets [30]. After each measurement, calibration films were irradiated in reference conditions with known doses. Approximately 24 h after dose delivery, both the measurement and calibration films were scanned together. A calibration curve was determined using all the calibration films of that measurement session, converting the optical density to absolute dose. After this, the optical density of the scanned film measurement was converted to dose, employing in-house developed software and all calibration films [31], [32]. Film and the online MRI were registered via point-matching MR markers of the film insert, visible in the online MRI to the central position of the circular cut-outs made in the film (see Figs. 3d and 3e). Subsequently, the transformation matrix of the registration between the film and online MRI was then applied to the dose file.

### 2.7 Analysis

The geometrical accuracy was obtained by fitting of the 3D planning dose file to the dosimeter’s dose readout (Fig. 3g). Fitting was performed using the Hooke–Jeeves direct search optimization method [33], with a correlation ratio as the similarity metric and a region of interest encapsulating both PTVs. After fitting, the geometrical shifts between the registered and fitted dose files were obtained for the x, y and z direction. 2D displacement vectors for the gel dosimeter were calculated in xy and yz planes for comparison to the two in-film plane measurements, as well as the 3D vectors. The reproducibility of the E2E test was assessed using the standard deviations calculated for all measurements of the respective dosimeter per measurement session. Dosimetric evaluation of the measurements was conducted using a global 3D gamma analysis, applying a 3%/3 mm criterion with a minimum dose threshold set at 20% of the maximum planned dose [34]. During this evaluation, the dose files were only registered to the dosimeter (Figs. 3c and 3f), and as such the geometrical inaccuracies were still present.

## 3 Results

**Fig. 4:**
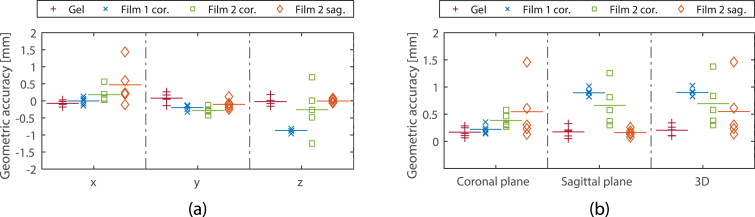
Geometric accuracy of measurement sessions one (gel and film 1 cor.) and two (film 2 cor. and film 2 sag.). In (a) the accuracy is shown for the x, y and z direction. In figure (b) the 2D coronal and sagittal, and the 3D displacement vectors are shown. The horizontal line depicts the mean value.

**Fig. 5:**
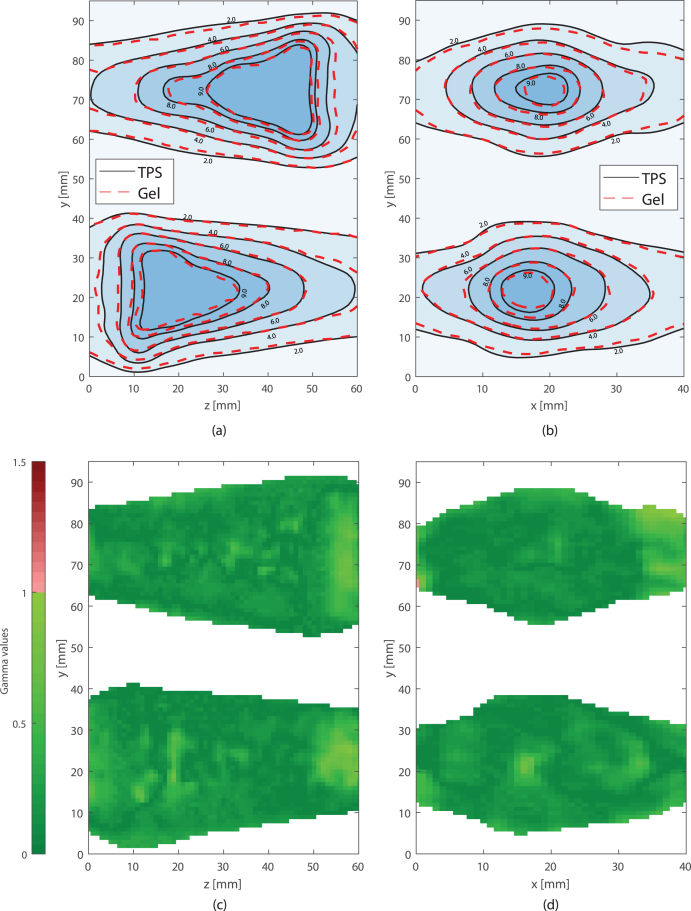
2D isodose lines for the sagittal (a) and coronal (b) plane of one of the gel dosimeter measurements and the planned dose. Numbers in the isodose lines are in Gy. γ maps are shown for the sagittal (c) and coronal (d) plane, using the same slice as in (a) and (b). White color represents the data not meeting the lower threshold imposed.

**Fig. 6:**
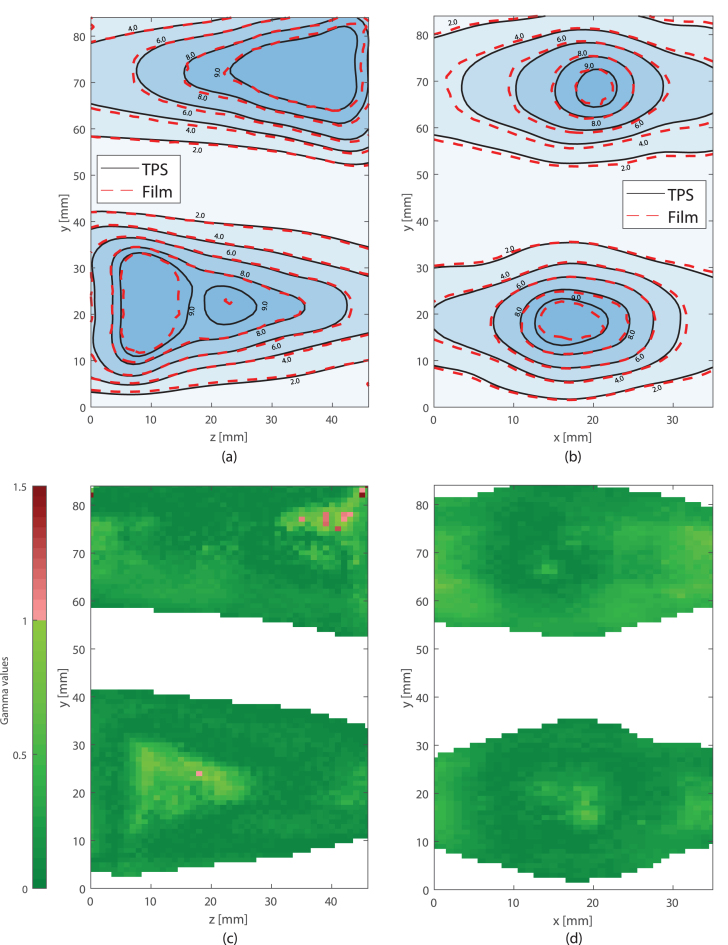
2D isodose lines for the sagittal (a) and coronal (b) plane measurements of film dosimeter and the planned dose. Numbers in the isodose lines are in Gy. Numbers in the isodose lines are in Gy. γ maps are shown for the sagittal (c) and coronal (d) plane, using the same slice as in (a) and (b). White color represents the data not meeting the lower threshold imposed.

The geometric accuracy and reproducibility of the gel dosimeters were high, and comparable to those found for the in-plane film dosimeters (see Fig. 4, Fig. 5, Fig. 6). The out-of-plane film measurements showed larger differences. Small differences between the absolute dose scaling for the film measurements of the second measurement session and the TPS were observed (see Fig. 6a). The average 1D (x, y and z directions) geometrical accuracy was ≤0.1mm and ≤0.3mm (see Supplementary S1) for the gel and in-plane film dosimeters, respectively.

For the gel measurements performed during the first measurement session, the geometric accuracy in the x, y, and z directions was high, with the largest deviation being 0.3 mm in the y-direction. The in-plane film measurements showed a maximum shift of 0.6 mm.

The average 2D vectors for the gel (in the xy and xz planes) were 0.2 ± 0.1 mm, and comparable to the in-plane film measurements. Furthermore, the average 3D vector found for the five measurements with the gel dosimeters were 0.2 ± 0.1 mm. All film measurements showed a lower geometric accuracy.

The mean gamma index pass rate for the gel measurements was 98.2% ± 1.1%. For the film measurements, the average pass rate was 97.4% ± 3.6%.

## 4 Discussion

In this study, we investigated the usability of gel dosimeters for the determination of the E2E geometrical accuracy of the online workflow on an MR-linac. 3D gel measurements were compared with 2D film dosimeter reference measurements, and showed to be suitable for E2E measurements on an MR-linac, with an average 3D geometrical accuracy of 0.2 mm.

Assessment of the 3D geometrical accuracy with film dosimeters was difficult, as indicated by the out-of-plane film measurements with larger average shifts and lower reproducibility. Notably, the coronal film measurements of sessions one and two yielded different results. These uncertainties were, however, within the voxel grid size used for the dose calculation. Overall, the 3D measured geometric uncertainties of the gel dosimeter were similar to the measured 2D in film-plane obtained geometric uncertainties.

The geometric uncertainties measured with gel dosimeters correspond well with the findings of previous studies that investigated the geometrical uncertainties of an MR-linac. During the first-in-man study of the Unity MR-linac by Raaymakers et al. geometrical acuracy was assessed both in a phantom and during patient treatments based on bony structures using MV projection images [4]. The geometrical accuracy was found to be better than 0.5 mm for phantom measurements and on average 0.3 mm during patient treatments. Another study, conducted by Bernchou et al. utilizing a 3D-printed film dosimetry phantom that hosts several structures, was used for a series of E2E tests on an MR-linac [13]. Their study showed similar geometrical uncertainties compared to this study for the ATS procedure, with an exception for the z-direction. Bernchou et al. reported a consistent offset of 0.8 ± 0.2 mm in the z-direction measured using in-plane film measurements, and were unable to identify the root cause. In this study, we did not find a shift in the z-direction for either the gel or in-plane film measurements.

During this study, a static, non-deforming phantom was used. Although the PTVs were rigidly propagated and do not represent a clinical treatment site, the measurements provide valuable information regarding the 3D geometrical accuracy that can be achieved by the MR-linac.

Gamma pass rates obtained during the measurements showed good agreement with the reference and were similar to those reported by Pappas et al. [16]. Additionally, all film measurements showed gamma passing rates comparable to the values found by Pappas et al. [16] and Raaymakers et al. [4]. For the second measurement session, conversion of the film proved to be more difficult, resulting in dose deviations in high dose regions, which resulted in a larger standard deviation for the film’s gamma pass rate.

The suitability of gel compared to other dosimeters such as film and 1D detector arrays, depends on their application. Film, for instance, is easier to store, can be processed in-house, and the resolution of the measurement plane is higher than what currently, reasonably, is obtainable with gel dosimeters. Additionally, with film dosimetry, the user has complete control over every aspect, from the initial measurement to the processing of the data. This level of control is not, reasonably, achievable with gel dosimetry. During this study, some of the film measurements showed minor deviations in the high dose regions as shown in Fig. 6a.

Nevertheless, both 2D film and 1D detector arrays lack high spatial 3D dose information. This becomes even more crucial when measuring treatment plans involving multiple targets [35], or multiple isocenters with abutting field segments [36], which require a detailed understanding of the 3D dose distribution. A 3D-measured dose distribution of an E2E test using gel dosimeters can provide the confirmation that machine and workflows are operating correctly. Additionally, for MR-linac sites with limited staff and local resources, the gel dosimeter can shipped back to the vendor for analysis.

In this study, we showed that gel dosimeters can be used for assessment of the end-to-end geometric accuracy of an MR-linac. The geometric accuracy and standard deviations of the gel dosimeter measurements were comparable to those obtained with in-plane film measurements. Gel dosimeters might be helpful for 3D dose assessment of various applications such as commissioning, end-to-end testing, investigating new treatment modalities, or auditing procedures.

## CRediT authorship contribution statement

**Stijn Oolbekkink:** Conceptualization, Methodology, Software, Investigation, Formal analysis, Validation, Data curation, Writing – original draft. **Jochem W.H. Wolthaus:** Conceptualization, Methodology, Software, Validation, Writing – review & editing, Supervision. **Bram van Asselen:** Conceptualization, Methodology, Validation, Writing – review & editing, Supervision. **Bas W. Raaymakers:** Conceptualization, Methodology, Validation, Writing – review & editing, Supervision, Funding acquisition.

## Declaration of competing interest

The authors declare the following financial interests/personal relationships which may be considered as potential competing interests: The authors acknowledge funding by the Dutch Research Council (NWO) through Project No. 18495 (ADEQUATE).

## Appendix A Supplementary data

MMC S1 A table showing the geometric accuracy for both dosimeters in the x, y, z, 2D, and 3D vectors is provided in the supplementary data.

## References

[1] Lagendijk J.J.W., Raaymakers B.W., Raaijmakers A.J.E., Overweg J., Brown K.J., Kerkhof E.M. (2008). MRI/linac integration. Radiother Oncol.

[2] Raaymakers B.W., Lagendijk J.J.W., Overweg J., Kok J.G.M., Raaijmakers A.J.E., Kerkhof E.M. (2009). Integrating a 1.5 T MRI scanner with a 6 MV accelerator: proof of concept. Phys Med Biol.

[3] Lagendijk J.J.W., Raaymakers B.W., van Vulpen M. (2014). The magnetic resonance imaging–linac system. Semin Radiat Oncol.

[4] Raaymakers B.W., Jürgenliemk-Schulz I.M., Bol G.H., Glitzner M., Kotte AN.T.J., van Asselen B. (2017). First patients treated with a 1.5 T MRI-Linac: clinical proof of concept of a high-precision, high-field MRI guided radiotherapy treatment. Phys Med Biol.

[5] Winkel D., Bol G.H., Kroon P.S., van Asselen B., Hackett S.S., Werensteijn-Honingh A.M. (2019). Adaptive radiotherapy: The Elekta Unity MR-linac concept. Clin Trans Radiat Oncol.

[6] Klein E.E., Hanley J., Bayouth J., Yin F.F., Simon W., Dresser S. (2009). Task Group 142 report: Quality assurance of medical accelerators. Med Phys.

[7] Seravalli E., van Haaren P.M.A., van der Toorn P.P., Hurkmans C.W. (2015). A comprehensive evaluation of treatment accuracy, including end-to-end tests and clinical data, applied to intracranial stereotactic radiotherapy. Radiother Oncol.

[8] Wang L., Kielar K.N., Mok E., Hsu A., Dieterich S., Xing L. (2012). An end-to-end examination of geometric accuracy of IGRT using a new digital accelerator equipped with onboard imaging system. Phys Med Biol.

[9] Molineu A., Followill D.S., Balter P.A., Hanson W.F., Gillin M.T., Huq M.S. (2005). Design and implementation of an anthropomorphic quality assurance phantom for intensity-modulated radiation therapy for the Radiation Therapy Oncology Group. Int J Radiat Oncol Biol Phys.

[10] Axford A., Dikaios N., Roberts D.A., Clark C.H., Evans P.M. (2021). An end-to-end assessment on the accuracy of adaptive radiotherapy in an MR-linac. Phys Med Biol.

[11] Elter A., Dorsch S., Mann P., Runz A., Johnen W., Spindeldreier C.K. (2019). End-to-end test of an online adaptive treatment procedure in MR-guided radiotherapy using a phantom with anthropomorphic structures. Phys Med Biol.

[12] Stark L.S., Andratschke N., Baumgartl M., Bogowicz M., Chamberlain M., Dal Bello R. (2020). Dosimetric and geometric end-to-end accuracy of a magnetic resonance guided linear accelerator. Phys Imaging Radiat Oncol.

[13] Bernchou U., Christiansen R.L., Bertelsen A., Tilly D., Riis H.L., Jensen H.R. (2021). End-to-end validation of the geometric dose delivery performance of MR linac adaptive radiotherapy. Phys Med Biol.

[14] Pappas E., Maris T., Angelopoulos A., Paparigopoulou M., Sakelliou L., Sandilos P. (1999). A new polymer gel for magnetic resonance imaging (MRI) radiation dosimetry. Phys Med Biol.

[15] Ibbott G.S., Le H.J., Roe Y. (2019). The MD Anderson experience with 3D dosimetry and an MR-linac. J Phys: Conference ser.

[16] Pappas E., Kalaitzakis G., Boursianis T., Zoros E., Zourari K., Pappas E.P. (2019). Dosimetric performance of the Elekta Unity MR-linac system: 2D and 3D dosimetry in anthropomorphic inhomogeneous geometry. Phys Med Biol.

[17] Deene Y.D., Wheatley M., Dong B., Roberts N., Jelen U., Waddington D. (2020). Towards real-time 4D radiation dosimetry on an MRI-Linac. Phys Med Biol.

[18] Roed Y., Ding Y., Wen Z., Wang J., Pinsky L., Ibbott G. (2017). The potential of polymer gel dosimeters for 3D MR-IGRT quality assurance. J Phys: Conference Ser.

[19] Lee H.J., Roed Y., Venkataraman S., Carroll M., Ibbott G.S. (2017). Investigation of magnetic field effects on the dose–response of 3D dosimeters for magnetic resonance – image guided radiation therapy applications. Radiother Oncol.

[20] Lee H.J., Kadbi M., Bosco G., Ibbott G.S. (2018). Real-time volumetric relative dosimetry for magnetic resonance—image-guided radiation therapy (MR-IGRT). Phys Med Biol.

[21] Han E.Y., Diagaradjane P., Luo D., Ding Y., Kalaitzakis G., Zoros E. (2020). Validation of PTV margin for Gamma Knife Icon frameless treatment using a PseudoPatient® Prime anthropomorphic phantom. J Appl Clin Med Phys..

[22] Makris D.N., Pappas E.P., Zoros E., Papanikolaou N., Saenz D.L., Kalaitzakis G. (2019). Characterization of a novel 3D printed patient specific phantom for quality assurance in cranial stereotactic radiosurgery applications. Phys Med Biol.

[23] Commission IE. IEC 61217 Radiotherapy equipment - Coordinates movements and scales. Geneva, Switzerland; 2011.

[24] Houweling A.C., van der Meer S., van der Wal E., Terhaard C.H.J., Raaijmakers C.P.J. (2010). Improved immobilization using an individual head support in head and neck cancer patients. Radiother Oncol.

[25] Pappas E., Angelopoulos A., Kipouros P., Vlachos L., Xenofos S., Seimenis I. (2003). Evaluation of the performance of VIPAR polymer gels using a variety of x-ray and electron beams. Phys Med Biol.

[26] Papadakis A.E., Maris T.G., Zacharopoulou F., Pappas E., Zacharakis G., Damilakis J. (2007). An evaluation of the dosimetric performance characteristics of N-vinylpyrrolidone-based polymer gels. Phys Med Biol.

[27] Pappas E. (2009). On the role of polymer gels in the dosimetry of small photon fields used in radiotherapy. J Phys: Conference Ser.

[28] Baldock C., Deene Y.D., Doran S., Ibbott G., Jirasek A., Lepage M. (2010). Polymer gel dosimetry. Phys Med Biol.

[29] Billas I., Bouchard H., Oelfke U., Duane S. (2019). The effect of magnetic field strength on the response of Gafchromic EBT-3 film. Phys Med Biol.

[30] Hackett S.L., van Asselen B., Wolthaus J.W.H., Kok J.G.M., Woodings S.J., Lagendijk J.J.W., al et. (2016). Consequences of air around an ionization chamber: Are existing solid phantoms suitable for reference dosimetry on an MR-linac?. Med Phys.

[31] Lewis D., Micke A., Yu X., Chan M.F. (2012). An efficient protocol for radiochromic film dosimetry combining calibration and measurement in a single scan. Med Phys.

[32] Lewis D., Devic S. (2015). Correcting scan-to-scan response variability for a radiochromic film-based reference dosimetry system. Med Phys.

[33] Hooke R., Jeeves T.A. (1961). Direct search solution of numerical and statistical problems. J ACM..

[34] Low D.A., Harms W.B., Mutic S., Purdy J.A. (1998). A technique for the quantitative evaluation of dose distributions. Med Phys.

[35] Lim S.B., Kuo L., Li T., Li X., Ballangrud A.M., Lovelock M. (2022). Comparative study of SRS end-to-end QA processes of a diode array device and an anthropomorphic phantom loaded with GafChromic XD film. J ACM.

[36] Chuter R.W., Brewster F., Retout L., Cree A., Aktürk N., Hales R. (2023). Feasibility of using a dual isocentre technique for treating cervical cancer on the 1.5 T MR-Linac. Phys Med Biol.

