# Supplementary material for: 3D gel dosimeter assessment for end-to-end geometric accuracy determination of the online adaptive workflow on the 1.5 T MR-linac
Source: Phys Imaging Radiat Oncol. 2024 Nov 5;32:100664. doi: 10.1016/j.phro.2024.100664 (PMC11585670; doi:10.1016/j.phro.2024.100664)
Supplement: MMC S1 — A table showing the geometric accuracy for both dosimeters in the x, y, z, 2D, and 3D vectors is provided in the supplementary data. [file mmc1.pdf]

3D gel dosimeter assessment for end-to-end geometric accuracy determination of the online adaptive workflow on the 1.5 T MR-linac

Stijn Oolbekkink[1], Jochem W. H. Wolthaus[1], Bram van Asselen[1] , Bas W. Raaymakers[1]
1) University Medical Center Utrecht, Heidelberglaan 100, 3584 CX, Utrecht, the Netherlands

Version typeset September 23, 2024

1 S1. Geometric accuracy

Table 1: Mean geometric uncertainty and standard deviations in millimeters of the measurements. Results showing or containing out-of-film-plane fitting directions are in italic font.

			x [mm]		y [mm]		z [mm]		2D [mm] (XY)		2D [mm] (YZ)		3D [mm]	
Session	Dosimeter	Plane	Mean	SD	Mean	SD	Mean	SD	Mean	SD	Mean	SD	Mean	SD
1	Gel	3D	-0.1	0.1	0.1	0.2	0.0	0.1	0.2	0.1	0.2	0.1	0.2	0.1
1	Film	Cor.	0.0	0.1	-0.2	0.1	*-0.9*	*0.1*	0.2	0.1	*0.9*	*0.1*	*0.9*	*0.1*
2	Film	Cor.	0.2	0.2	-0.3	0.1	*-0.3*	*0.7*	0.4	0.1	*0.7*	*0.4*	*0.7*	*0.4*
2	Film	Sag.	*0.5*	*0.6*	-0.1	0.1	0.0	0.1	*0.5*	*0.5*	0.2	0.1	*0.5*	*0.5*
